# Basaloid squamous cell carcinoma of the maxillary gingiva: A case report and review of the literature

**DOI:** 10.3892/ol.2014.2318

**Published:** 2014-07-04

**Authors:** SHULE XIE, MARIUS BREDELL, HONGYU YANG, SHIYUE SHEN, HUIJUN YANG

**Affiliations:** 1 Department of Oral and Maxillofacial Surgery, Peking University, Shenzhen Hospital, Shenzhen, Guangdong 518001, P.R. China; 2 Department of Cranio-Maxillofacial and Oral Surgery, University Hospital of Zürich, Zürich CH-8091, Switzerland

**Keywords:** basaloid squamous cell carcinoma, gingival carcinoma, squamous cell carcinoma, immunohistochemistry

## Abstract

Basaloid squamous cell carcinoma (BSCC) is a rare, but distinct histologic variant of squamous cell carcinoma in the head and neck region. It is considered to have a poor prognosis due to its aggressive behavior and tendency to metastasize. The usual sites of BSCC are the floor of the mouth, hypopharynx and base of the tongue, and according to the English-language literature its presentation in the gingiva is somewhat uncommon. In the current report, the unusual case of a 40-year-old male is presented; the patient exhibited a painless irregular mass in the maxillary gingiva, which infiltrated the maxillary sinus, as observed by computed tomography. Hematoxylin and eosin-stained sections revealed a diagnosis of BSCC with typical central necrosis in the cancer nests, which contained basaloid and squamous cells. Immunohistochemistry revealed that p63 was weakly positive, high molecular weight cytokeratin (CK) was focally positive, and S-100, CK7, CK14 and vimentin were negative. It must be noted that histopathology results may be incorrectly interpreted as adenoid cystic carcinoma, undifferentiated carcinoma and basal cell adenocarcinoma.

## Introduction

Basaloid squamous cell carcinoma (BSCC) is an uncommon variant of squamous cell carcinoma (SCC), which was first described by Wain *et al* (1) in 1986. BSCC generally present in the upper aerodigestive tract, particularly in the larynx, hypopharynx and the base of the tongue (2). Ide *et al* (3) reported 46 cases of BSCC in the oral mucosa, where only one case occurred in the gingival tissues. In addition, Hirai *et al* (4) described two cases of BSCC in the mandibular gingiva. In the current study, an additional case of BSCC in the maxillary gingiva is presented and the clinical features of BSCC are reviewed according to the current literature. Patient provided written informed consent.

## Case report

**Figure 1:**
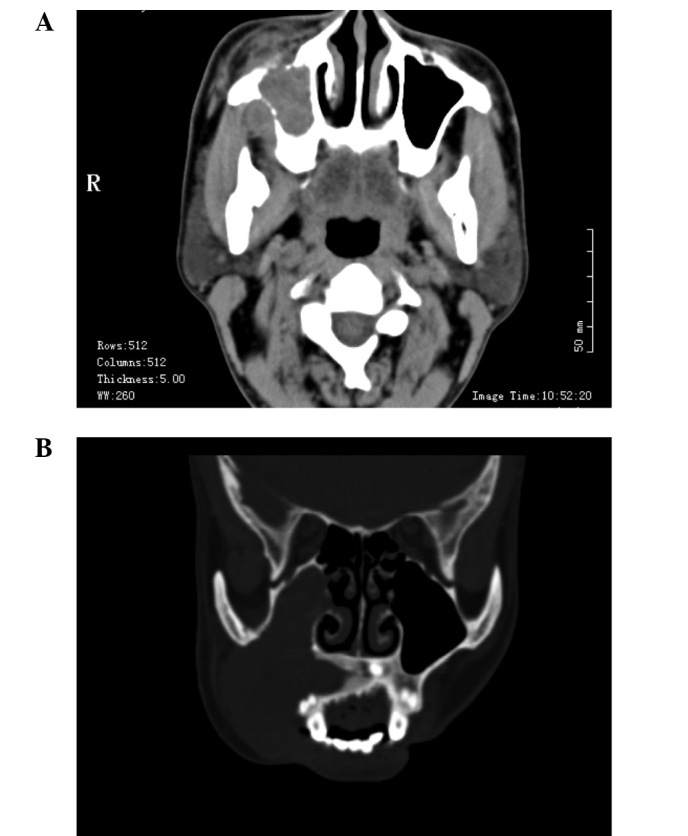
Computed tomography images of the basaloid squamous cell carcinoma. (A and B) The mass invaded the maxillary sinus and the central region of the hard palate.

In October 2009, a 40-year-old male visited the outpatient clinic of Peking University, Shenzhen Hospital (Shenzhen, China) presenting with a painless irregular mass of the right maxillary gingiva as well as nasal obstruction following the presentation of the initial symptoms two months previously. The patient’s medical history was unremarkable. The patient had a history of smoking for a period of 15 years (frequency, 10/day), however, denied excessive alcohol consumption. The intraoral examination revealed a gray, irregular mass (size, 3×2.5×2 cm) in the right maxillary posterior buccal gingiva, which elicited marginal pain on palpation. An extraoral clinical examination identified various palpable, mobile lymph nodes in the right submandibular region, measuring ~1×1×1 cm that were firm and non-tender on palpation. A computed tomography (CT) scan demonstrated a tumor, which involved the right maxillary sinus and infiltrated the central region of the hard palate (Fig. 1). The chest CT was negative for distant metastatic lesions. The lesion was clinically and radiologically classified as cT4 cNx cM0, according to the American Joint Committee on Cancer (AJCC) staging manual (5). The treatment comprised of an extended surgical excision of the tumor, which involved a partial maxillectomy with mandibulectomy and ipsilateral functional neck dissection at levels I-III, where four enlarged lymph nodes were removed at level Ib.

To investigate the excised tissue, the tissues were fixed in 10% buffered formalin and processed via the usual methods for paraffin embedding. The paraffin sections were stained with hematoxylin and eosin. Immunohistochemical staining was performed using the BOND-MAX automated immunostainer (Vision BioSystems, Melbourne, Australia) to further define the diagnosis of the tumor. The antibodies that were used for immunohistochemistry are presented in Table I. The antigen retrieval method was heat-induced epitope retrieval in EDTA at an alkaline pH (pH 8.0). Adequate positive and negative tissue controls were used.

**Figure 2:**
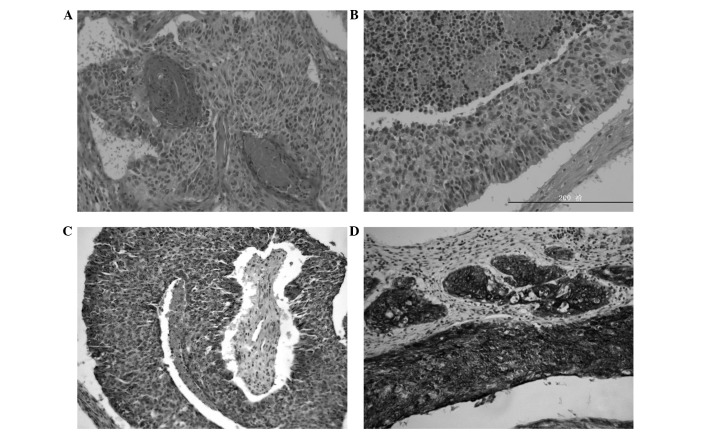
(A) Histological sections of the basaloid squamous cell carcinoma (hematoxylin and eosin [H&E] stain; magnification, ×200) displayed central comedo-type necrosis in the cancer nest and (B) peripheral palisading with radially arranged cells at the periphery of the lobules. (C) Diffuse tumor cells were weakly positive for the p63 marker, and (D) intense high molecular weight cytokeratin-positivity was observed in the membrane and cytoplasm of the cancer cells (B–D: H&E stain; magnification, ×200).

Microscopically, the tissues from the primary site demonstrated that there were two cellular populations; epithelial-like and basaloid tumor cells. The basaloid cells formed the primary invasive component of the cancer nests and were arranged in cords, trabeculae and lobules that occasionally demonstrated a pseudoglandular formation. These cells exhibited peripheral palisading and hyperchromatic nuclei with a high nucleus-cytoplasm ratio as well as a scant cytoplasm (Fig. 2A and B). Mitotic figures were observed within the nests and necrotic foci were scattered throughout the visual field. Components of SCC exhibiting keratinization were scarce, which differs to the features of conventional SCC. Immunohistochemically, the basaloid carcinoma cells were weakly positive for p63 (Fig. 2C) and focally positive for high molecular weight cytokeratin (CK-H; Fig. 2D), and negative for cytokeratin (CK)7, CK14, S-100 protein and vimentin. According to the clinical presentation, histopathological features and immunohistochemical findings, a final diagnosis of BSCC in the maxillary gingiva was determined. The surgical excision margins were healthy and the neck lymph node histopathology did not reveal any positive lymph nodes. The pathological staging of pT4 pN0 pM0 was established according to the AJCC staging manual.

However, the patient experienced recurrence half a year later and presented with a painful mass of the right cheek without enlarged lymph nodes on palpation. Following a failed tumor response to chemotherapy (dose, 16 mg pingyangmycin per day for seven days) and dimensional conformal radiotherapy (dose, 50 Gy), a second surgical intervention comprising of an extended resection of the neoplasm was conducted. Three years following treatment the patient remains free from tumor recurrence.

## Discussion

BSCC is a rare and malignant tumor that presents in the head and neck region, including the oral mucosa, and has been defined as an aggressive and distinct variant of SCC, which is composed of basaloid and squamous components, according to the World health Organization (6). BSCC is particularly uncommon in the oral cavity and more so in the gingiva. According to Hirai *et al* (4), only eight cases of BSCC in the gingiva have been reported in the English literature (4,7–9), with only one case of BSCC occurring in the maxillary gingiva.

The clinical features of the BSCC cases that presented in the gingiva are reviewed and summarized in Table II. Two patients were female and seven were male with an age range of 40–79 years (mean age, 60.1 years). The most frequent site of origin was the mandible (n=7) followed by the maxilla (n=2). According to the standard tumor-node-metastasis (TNM) staging, provided by the AJCC, three patients presented in stage I, two in stage II, three in stage III, and one in stage IV. All of the patients were treated using surgery, four underwent neck dissections and three received adjuvant radiotherapy. Five patients had survived at the median follow-up time of 56 months.

The prognosis of patients with BSCC compared with patients with conventional SCC remains uncertain. Winzenburg *et al* (10) first identified that distant metastases occurred in 52% of patients with BSCC and in 13% of patients with poorly differentiated SCC. Soriano *et al* (11) showed that patients with SCC were associated with notably higher survival rates when compared with patients with BSCC; furthermore, the rate of distant metastasis was six times higher in the cases of BSCC. However, de Sampaio Góes *et al* (12) declared that the prognosis did not differ between patients with BSCC of the oral cavity and those with conventional SCC.

The diagnosis of BSCC is currently based on histological criteria, including focal squamous differentiation, a basaloid pattern that is associated with frank invasive SCC or carcinoma *in situ*. However, the histopathological diagnosis of BSCC is difficult to differentiate from that of adenoid cystic carcinoma (ACC), poorly differentiated carcinoma and basal cell adenocarcinoma. BSCC may easily be misdiagnosed as ACC, particularly when a small biopsy sample is used.

Previous studies have advocated that immunohistochemical markers, including CK7, CK14, p63, CK-H, S-100 and vimentin aid with distinguishing BSCC from ACC (13,14). Colleta *et al* (13) reported that the majority of cancer cells of ACC express CK7, which indicates a ductal-pattern possibly of salivary gland origin, while in BSCC, the basaloid cells exhibit positive expression for CK17 and negative expression for vimentin, S-100, CK7, CK8 or CK20. The p63 staining pattern of BSCC and ACC is markedly different; the positive p63 staining is diffused in ~100% of the BSCC cancer cells, while ACC demonstrates a compartmentalized pattern within the tumor nests (15). In addition, the expression of CK-H is positive in BSCC cases (14). In the present case the immunohistochemical staining was positive for p63 and CK-H, and negative for S-100, vimentin, CK14 and CK7. Thariat *et al* (16) also advocated another criterion (in addition to the original criteria and immunohistochemical findings reported by Wain *et al* [1]) and proposed that, owing to its dual behavior that is marginally dependent on its association with the human papilloma virus (HPV), the BSCC patients should be systematically examined for their HPV status as this may determine the treatment response (17).

BSCC require aggressive multimodality treatment, including radical surgical excision, neck dissection, radiotherapy and regular chemotherapy due to the high overall mortality rate. Although chemotherapy is recommended by certain authors due to the high incidence of distant metastasis and the relatively poor prognosis (17), a standard chemotherapy regimen for BSCC has not yet been established. Furthermore, investigation of a greater number of patients is required to determine the efficacy of chemotherapy for BSCC of the head and neck. Bonner *et al* (18) advocated that immunotherapy elicited an improved treatment effect when compared with radiotherapy alone and resulted in a reduced mortality rate.

In conclusion, the aggressive behavior of BSCC has been presented using a rare case of the maxillary gingiva. The potential difficulties of a histological diagnosis were discussed and the possible obstacles to an accurate diagnosis were emphasized. Further studies with uniform reporting are required in order to optimize the establishment of a diagnosis and define the optimum treatment strategies.

## Figures and Tables

**Table I:** Primary antibodies adopted for immunohistochemical staining.

Antibody	Clone	Source	Dilution	Result
p63	4A4	Santa Cruz^a^	1:75	+
CK7	OV-TL12/30	Dako^b^	1:50	−
CK14	LL002	Novocastra^c^	1:50	−
CK-H	34βE12	Dako^b^	1:50	+
Vimentin	V9	Dako^b^	1:50	−
S-100	Antiserum	Dako^b^	1:4,000	−

a Santa Cruz Biotechnology, Inc. (Santa Cruz, CA, USA);

b Dako (Carpinteria, CA, USA);

c Novocastra Laboratories (Newcastle, UK).

CK, cytokeratin; CK-H, high molecular weight cytokeratin.

**Table II:** Reported cases of BSCC of the gingiva.

First author (ref)	Year	Age/gender	Stage	Location of lesion	Treatment	Final outcome	Follow-up period, months
Wedenberg *et al* (7)	1997	55/M	I	Maxilla	S	A	5
Abiko *et al* (8)	1998	79/F	I	Mandible	S	A	24
Yu *et al* (9)	2008	61/M	II	Mandible	S + FND	A	120
Yu *et al* (9)	2008	56/M	IV	Mandible	S	D	180
Yu *et al* (9)	2008	65/M	III	Mandible	S	D	2.5
Subramania *et al* (10)	2009	72/F	III	Mandible	S + FND + RT	A	12
Hirai *et al* (4)	2009	55/M	II	Mandible	S + FND	A	79
Hirai *et al* (4)	2009	65/M	I	Mandible	S + RT	A	60
Present case	2010	40/M	III	Maxilla	S + FND + RT	A	25

S, surgery; FND, functional neck dissection; RT, radiotherapy; A, alive; D, died.

## Abbreviations

BSCC: basaloid squamous cell carcinoma
SCC: squamous cell carcinoma
ACC: adenoid cystic carcinoma
CK: cytokeratin
